# The impact of socioeconomic status on glioma survival: a retrospective analysis

**DOI:** 10.1007/s10552-025-01960-1

**Published:** 2025-01-19

**Authors:** Maria Söderlund, Carl Almqvist, Olle Sjöström, Anna M. Dahlin, Sara Sjöström, Barbro Numan Hellquist, Beatrice Melin, Maria Sandström

**Affiliations:** Department of Diagnostics and Intervention, Oncology, Umeå University, SE-901 87, Umeå, Sweden

**Keywords:** Glioma, Survival, Socioeconomic status, Education level, Travel time, Cohabitation status, Region of residence

## Abstract

**Purpose:**

Although sociodemographic factors such as socioeconomic status (SES), travel time to health care, cohabitation status, and region of residence are observed to influence incidence and survival for several types of cancers, it is unclear whether similar effects have been observed in patients with glioma. This study investigates whether these factors affect survival for glioma patients.

**Methods:**

In this retrospective study, the Swedish National Quality Registry for Brain Tumors was used to identify 1,276 patients with glioma WHO grade I–IV for whom data were deposited between 2009 and 2013. The RISK North database, which links data from the National Cancer Quality Register with citizen demographic data from the Longitudinal Integration Database for Health Insurance and Labor Market Studies (LISA), the Total Population Registry (TPR), and the Geography Database (GD), was utilized to assess survival in patients with glioma in relation to education level, cohabitation status, travel time to regional hospitals, and region of residence.

**Results:**

In the multivariable analysis, longer survival was observed among WHO grade III-IV glioma patients with higher education level (middle school (ref) HR: 1, high school HR: 0.81 CI [0.67–0.98], *p* = 0.033; university/college HR: 0.81 CI [0.66–1.00], *p* = 0.048). Survival was not associated with travel time, cohabitation status, or region of residence in the multivariable survival analysis.

**Conclusion:**

Low education level was associated with reduced survival for patients with glioma WHO grade III and IV in multivariable survival analyses, but no differences in survival were found in relation to travel time, cohabitation status, or region of residence.

**Supplementary Information:**

The online version contains supplementary material available at 10.1007/s10552-025-01960-1.

## Introduction

Brain tumors are classified into different tumor entities through histological and molecular parameters according to the 2021 World Health Organization (WHO) Classification of Tumors of the Central Nervous System [1]. Gliomas are categorized as low-grade (grade 1–2) or high-grade (grade 3–4). Glioblastoma (WHO grade 4) is the most common and most malignant glioma.

There is evidence to suggest that some nonbiological indicators predict cancer incidence and outcomes. Socioeconomic status (SES), for which level of education is commonly used as a proxy, often contributes to health disparities [2]. Some studies report that high SES is associated with a higher incidence of glioma [3, 4], but others have not found an association [5, 6]. Earlier studies indicate that SES is associated with the type of treatment [7] and survival for glioma patients [5, 8].

Another potential factor influencing treatment and survival is the travel time taken to receive health care services. Travel burden for receiving treatment has been described for several cancers [9], but not for glioma, and it has been found that travel time might affect treatment choices and participation in clinical studies [10, 11]. Walker et al. found small prognostic differences for patients living in rural or urban areas [8]. However, the relation of travel time with treatment and survival has not been extensively studied in glioma patients.

Patients with brain tumors commonly have disabilities affecting cognition and decision making and are often dependent on help from others. For health care providers, it is therefore important to take the patients’ network into consideration. Being a cohabitant (i.e., living together with someone) has been investigated as a potential factor influencing cancer outcomes. One study showed that marital status was associated with earlier stage at diagnosis, a larger proportion of treatment with curative intent, and better disease-specific survival for patients with several types of cancer, but not including brain tumors [12]. In patients with central nervous system (CNS) tumors, Dalton et al. found no difference in relative survival between married and divorced patients [5]. For patients with glioblastoma, Xie et al. found marriage to be associated with a survival advantage [13].

Finally, in the Swedish context, the potential relation between region of residence and cancer outcomes has been investigated. Differences between Swedish regions in incidence and survival have been described for several types of cancer but data were not reported for brain tumor patients separately [14]. The North differs from the southern regions, as it has lower population density, and longer travel distances to health care services. The education level is higher in the southern regions than in the North [15]. This variation in sociodemographic factors increases the importance of the regional comparison.

This study investigates whether SES (estimated through highest completed level of education), travel time to the closest regional hospital, and cohabitation status affect the survival for patients with glioma and whether there are differences in survival between the North and three regions in southern Sweden.

## Materials and methods

This retrospective observational study included all patients diagnosed with glioma WHO grade I–IV registered in the National Quality Registry for Brain Tumors between 2009 and 2013 in the North of Sweden and the three southern regions with high registry coverage. The glioma patients in this study were diagnosed before 2013, and the tumors were therefore classified according to the 2007 WHO Classification, where tumor grade is written in Roman numerals (I–IV) [16].

### Data sources and retrieval

The National Quality Registry for Brain Tumors records primary brain tumors (except CNS lymphoma) in adults. The coverage of the quality registry is evaluated by the Regional Cancer Centres in Sweden, by comparing it with the National Cancer Registry, where it is mandatory to register all detected cases of cancer. The coverage was 98–100% in the southern regions (Middle Sweden, Stockholm-Gotland, and Southeastern) and 97–100% in the North between 2009 and 2013. The health care regions South and West had lower coverage (South: 4–79%, West: 88–98%). Therefore, data from the health care regions South and West were excluded from the analysis, following the approach taken by similar previous analyses using this registry [17, 18]. For this study, cases were selected using International Statistical Classification of Diseases and Related Health Problems (ICD)-7 code 193 (malignant neoplasm of the brain and other parts of the nervous system) [19]. To identify patients with glioblastoma, Systematized Nomenclature of Medicine (SNOMED) code 94,403 was used. The date of diagnosis was defined as the date of the pathology report. Surgery was categorized as biopsy or resection. In case of resection, a judgment was made from the postoperative magnetic resonance imaging performed within 48 h after surgery as gross total or partial resection. Grade was obtained from the pathology report. Age at diagnosis, sex, smoking status, WHO performance status (PS) [20], and type of surgery were retrieved from the quality registry. The data on WHO PS and smoking were incomplete (32% missing for WHO PS and 30% for smoking), and those variables were therefore omitted from further analysis.

The RISK North database was constructed to study cancer treatment and survival in relation to sociodemographic factors in the Swedish population [21]. This database combines data from several health care and demographic registries through the unique personal identity numbers that are assigned to all Swedish residents. In this study, data originating from the following sources were retrieved from the RISK North; the National Quality Registry for Brain Tumors, data on education level were retrieved from the Longitudinal Integration Database for Health Insurance and Labor Market Studies (LISA), data on cohabitation status were obtained from the Total Population Registry (TPR), and the Geography Database (GD) was used to obtain GPS coordinates for the patients’ home address. The TPR encompasses all inhabitants in Sweden and contains data on births, death, marital status, and migration [22], the LISA registers information on occupation, education, and income for the adult Swedish population [23], and the GD registers the geographical coordinates of the home address for all persons residing in Sweden.

### Sociodemographic covariates

SES was defined using the highest completed level of education as a proxy. Education level was divided into three categories: middle school (up to 9 years of compulsory school), high school (secondary education of 2 or 3 years), and university/college. According to the International Standard Classification of Education (ISCED) 2011 that is commonly used for categorizing education level, middle school corresponds to ISCED 0–2, high school to ISCED 2–3, and university/college to ISCED 4–8 [24]. Cohabitation status was defined as living alone versus living with another adult. Travel time by car from the patient’s home address to the regional hospital was estimated based on GPS coordinates using ArcGIS® Pro (2.1.2) and ArcGIS online (Esri, Redlands, CA, USA) and was categorized into ten-minute intervals. The regional hospital was defined as the closest hospital equipped to treat brain tumors (i.e., the closest hospital with a neurosurgical unit) in the health care region. The northern Swedish health care region (the North) consists of the four northernmost county councils (Norrbotten, Västerbotten, Jämtland-Härjedalen, and Västernorrland). In this study, the Southern regions were defined as a group including the health care regions Middle Sweden, Stockholm-Gotland, and Southeastern.

### Statistical analyses

The analyses were conducted with the study patients classified into WHO grade I–II, WHO grade III–IV, and WHO grade IV only (glioblastoma) subgroups. To test for differences in proportions, Pearson’s chi-square test was used. To compare differences in means between groups, analysis of variance (ANOVA) was used. The type I error rate was set at 5%, and all tests were two-tailed. Cox proportional hazard analysis was used to estimate hazard ratios (HRs) with 95% confidence intervals (CIs) in a multivariable survival analysis of the association between survival, education level, travel time, cohabitation status, and region of residence considering the potential confounders sex, age at diagnosis, and extent of surgery. Survival time was calculated from the date of diagnosis to the date of death or last follow-up (2014-12-31). Statistical analyses were performed using R, version 3.6.0 (R Core Team, Vienna, Austria) [25].

## Results

### Characteristics of the study patient cohort

The population in the southern regions was larger than that in the North during the study period (4,951,823 vs. 878,706 as of 2013) (Online Resource 1). For the study cohort, we identified 1,276 patients with glioma WHO grade I–IV from the National Quality Registry for Brain Tumors (2009–2013). In total, 799 patients with glioblastoma, 214 patients with glioma WHO grade III, and 263 patients with glioma WHO grade I–II were identified and included in subsequent analyses (Table 1).

**Table 1:** Characteristics of patients with glioma categorized by the WHO grading system

Variable	Level	Glioma WHO grade I–II			Glioma WHO grade III–IV			Glioblastoma (Glioma WHO grade IV)		
		Northern Sweden	Southern regions	Total	Northern Sweden	Southern regions	Total	Northern Sweden	Southern regions	Total
Total	*n*	50	213	263	209	804	1,013	176	623	799
Sex										
Male	*n* (%)	24 (48)	110 (52)	134 (51)	134 (64)	487 (61)	621 (61)	108 (61)	382 (61)	490 (61)
Female	*n* (%)	26 (52)	103 (48)	129 (49)	75 (36)	317 (39)	392 (39)	68 (39)	241 (39)	309 (39)
Missing	*n* (%)	0 (0)	0 (0)	0 (0)	0 (0)	0 (0)	0 (0)	0 (0)	0 (0)	0 (0)
*Chi*^*2*^*-test*^*a*^	*p*		*0.76*			*0.39*			*1.00*	
Age at diagnosis (years)										
18–39	*n* (%)	18 (36)	78 (37)	96 (36)	8 (4)	50 (6)	58 (6)	5 (3)	21 (3)	26 (3)
40–59	*n* (%)	15 (30)	87 (41)	102 (39)	53 (25)	275 (34)	328 (32)	41 (23)	210 (34)	251 (31)
60–69	*n* (%)	10 (20)	33 (15)	43 (16)	71 (34)	270 (34)	341 (34)	62 (35)	219 (35)	281 (35)
≥ 70	*n* (%)	7 (14)	15 (7)	22 (8)	77 (37)	209 (26)	286 (28)	68 (39)	173 (28)	241 (30)
Missing	*n* (%)	0 (0)	0 (0)	0 (0)	0 (0)	0 (0)	0 (0)	0 (0)	0 (0)	0 (0)
*Chi*^*2*^*-test*^*a*^	*p*		*0.25*			*0.006*			*0.02*	
Extent of surgery										
Gross total resection	*n* (%)	16 (32)	98 (46)	114 (43)	42 (20)	331 (41)	373 (37)	38 (22)	273 (44)	311 (39)
Partial resection	*n* (%)	17 (34)	81 (38)	98 (37)	78 (37)	248 (31)	326 (32)	68 (39)	186 (30)	254 (32)
Biopsy	*n* (%)	17 (34)	33 (15)	50 (19)	89 (43)	188 (23)	277 (27)	70 (40)	133 (21)	203 (25)
Unknown	*n* (%)	0 (0)	1 (0.5)	1 (0)	0 (0)	37 (5)	37 (4)	0 (0)	31 (5)	31 (4)
Missing	*n* (%)	0 (0)	0 (0)	0 (0)	0 (0)	0 (0)	0 (0)	0 (0)	0 (0)	0 (0)
*Chi*^*2*^*-test*^*a*^	*p*		*0.02*			< *0.001*			< *0.001*	
Education level										
Middle school	*n* (%)	14 (28)	31 (15)	45 (17)	56 (27)	202 (25)	258 (25)	49 (28)	162 (26)	211 (26)
High school	*n* (%)	20 (40)	86 (40)	106 (40)	94 (45)	322 (40)	416 (41)	74 (42)	245 (39)	319 (40)
University/college	*n* (%)	15 (30)	92 (43)	107 (41)	58 (28)	274 (34)	332 (33)	52 (30)	212 (34)	264 (33)
Missing	*n* (%)	1 (2)	4 (2)	5 (2)	1 (0.5)	6 (1)	7 (1)	1 (0.6)	4 (0.6)	5 (1)
*Chi*^*2*^*-test*^*a*^	*p*		*0.05*			*0.2*			*0.53*	
Travel time (min)										
< 30	*n* (%)	7 (14)	105 (49)	112 (43)	19 (9)	385 (48)	404 (40)	14 (8)	299 (48)	313 (39)
30–59	*n* (%)	0 (0)	39 (18)	39 (15)	4 (2)	159 (20)	163 (16)	4 (2)	124 (20)	128 (16)
60–119	*n* (%)	8 (16)	40 (19)	48 (18)	32 (15)	131 (16)	163 (16)	28 (16)	105 (17)	133 (17)
≥ 120	*n* (%)	35 (70)	29 (14)	64 (24)	154 (74)	129 (16)	283 (28)	130 (74)	95 (15)	225 (28)
Missing	*n* (%)	0 (0)	0 (0)	0 (0)	0 (0)	0 (0)	0 (0)	0 (0)	0 (0)	0 (0)
*Chi*^*2*^*-test*^*a*^	*p*		< *0.001*			< *0.001*			< *0.001*	
Cohabitation status										
Living alone	*n* (%)	10 (20)	71 (33)	81 (31)	58 (28)	238 (30)	296 (29)	51 (29)	183 (29)	234 (29)
Not living alone	*n* (%)	40 (80)	142 (67)	182 (69)	151 (72)	566 (70)	717 (71)	125 (71)	440 (71)	565 (71)
Missing	*n* (%)	0 (0)	0 (0)	0 (0)	0 (0)	0 (0)	0 (0)	0 (0)	0 (0)	0 (0)
*Chi*^*2*^*-test*^*a*^	*p*		*0.1*			*0.66*			*0.99*	
WHO PS^b^										
0	*n* (%)	16 (32)	88 (41)	104 (40)	28 (13)	249 (31)	277 (27)	25 (14)	182 (29)	207 (26)
1	*n* (%)	4 (8)	31 (15)	35 (13)	41 (20)	143 (18)	184 (18)	34 (19)	114 (18)	148 (19)
2	*n* (%)	0 (0)	14 (7)	14 (5)	38 (18)	128 (16)	166 (16)	35 (20)	101 (16)	136 (17)
3	*n* (%)	1 (2)	1 (0.5)	2 (1)	14 (7)	50 (6)	64 (6)	12 (7)	43 (7)	55 (7)
4	*n* (%)	1 (2)	2 (1)	3 (1)	4 (2)	14 (2)	18 (2)	2 (1)	11 (2)	13 (2)
Missing	*n* (%)	28 (56)	77 (36)	105 (40)	84 (40)	220 (27)	304 (30)	68 (39)	172 (28)	240 (30)
*Chi*^*2*^*-test*^*a*^	*p*		*0.22*			*0.02*			*0.001*	
Smoking										
Never smoker	*n* (%)	26 (52)	114 (54)	140 (53)	96 (46)	358 (45)	454 (45)	80 (45)	278 (45)	358 (45)
Ex-smoker	*n* (%)	6 (12)	18 (8)	24 (9)	35 (17)	106 (13)	141 (14)	29 (16)	79 (13)	108 (14)
Current smoker	*n* (%)	5 (10)	23 (11)	28 (11)	24 (11)	82 (10)	106 (10)	22 (13)	67 (11)	89 (11)
Missing	*n* (%)	13 (26)	58 (27)	71 (27)	54 (26)	258 (32)	312 (31)	45 (26)	199 (32)	244 (31)
*Chi*^*2*^*-test*^*a*^	*p*		*0.89*			*0.27*			*0.30*	

^a^The chi^2^-test was used to assess differences between northern Sweden and the three southern regions

^b^WHO PS = WHO performance status

### Multivariable survival analyses show better survival for patients with higher education level in glioma WHO grade III-IV but no differences for cohabitation status, travel time, or region

**Fig. 1:**
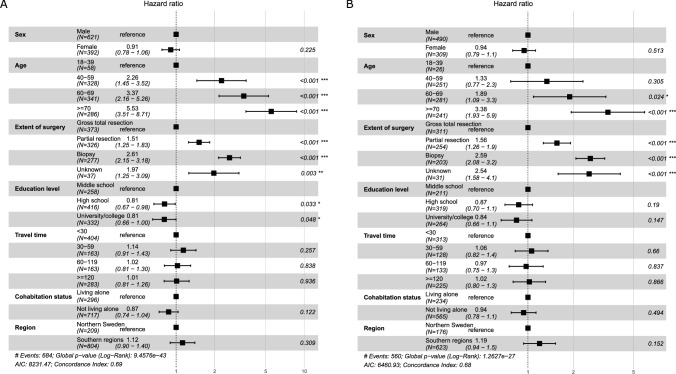
Cox regression analysis of factors related to overall survival in 1013 patients with WHO grade III–IV glioma (**A**) and 799 patients with glioblastoma (**B**)

A hazard analysis was conducted to determine whether the sociodemographic variables included in this study influence the survival of patients with glioma. In the multivariable model, survival in patients with glioma WHO grade III–IV was significantly longer among patients with higher education level (middle school (ref) HR: 1; high school HR: 0.81 CI [0.67–0.98], *p* = 0.033; university/college HR: 0.81 CI [0.66–1.00], *p* = 0.048) but did not differ by travel time, cohabitation status, or region of residence (Fig. 1A).

For the glioblastoma subgroup, survival was significantly shorter for patients with older age at diagnosis (18–39 years (ref) HR: 1; 40–59 years HR: 1.33 CI [0.77–2.30] *p* = 0.305; 60–69 years HR: 1.89 CI [1.09–3.30] *p* = 0.024; ≥ 70 years HR: 3.38 CI [1.93–5.90], *p* < 0.001) and for patients with less extensive surgery (gross total resection (ref) HR: 1; partial resection HR: 1.56 CI [1.26–1.90], *p* < 0.001; biopsy HR: 2.59 CI [2.08–3.20], *p* < 0.001). In survival analysis for the glioblastoma subgroup, a similar inverse association by education level was noted as for grade III-IV but it was not statistically significant. Estimates for travel time, cohabitation status, or region of residence were not statistically significant. (Fig. 1B).

**Fig. 2:**
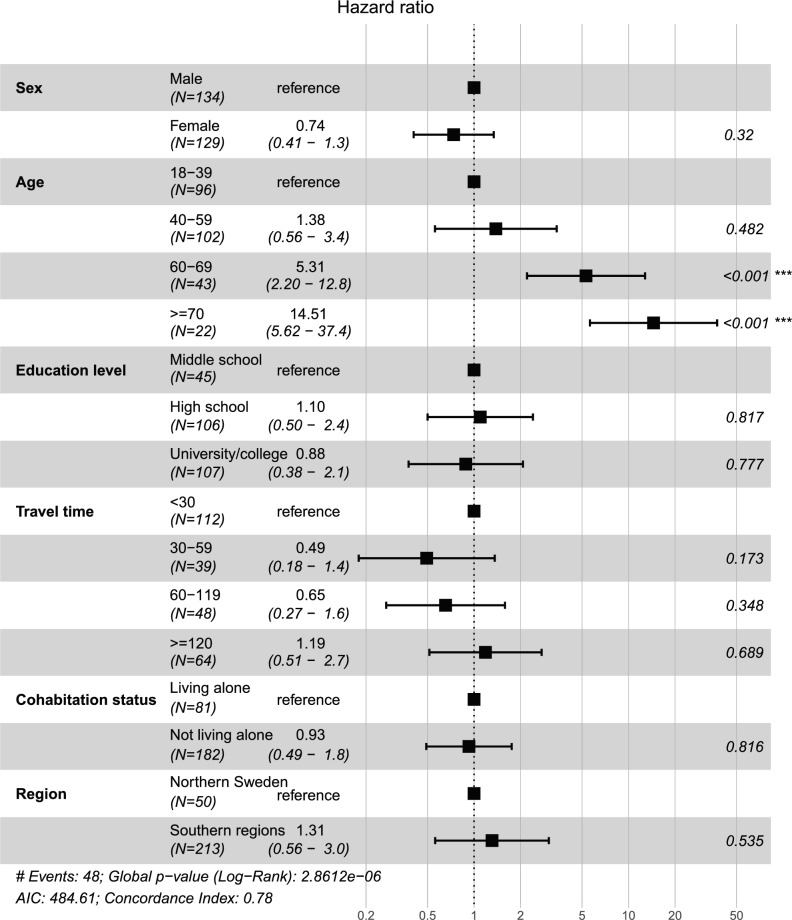
Cox regression analysis of factors related to overall survival in 263 patients with glioma WHO grade I–II

For patients with glioma WHO grade I–II, older age at diagnosis was associated with shorter survival. In the same analysis, education level, travel time, cohabitation status, and region of residence were not associated with survival (Fig. 2).

### Patients with glioma WHO grade III-IV living in the North were older, had less extensive surgery and longer distance to a regional hospital

A comparison between the regions was made regarding sociodemographic factors to study regional differences. In the glioma WHO grade III–IV subgroup and the glioblastoma subgroup, the age distribution differed between the regions, with the North having larger proportions of patients in the older age groups (*p* = 0.006 for the glioma WHO grade III–IV subgroup; *p* = 0.02 for the glioblastoma subgroup). In the subgroup of patients with glioma WHO grade I–II, the age distribution did not differ between regions (Table 1).

In terms of the extent of surgery, it was more common for patients in the North to receive less extensive surgery, with 40% of patients with glioblastoma receiving a biopsy as the only surgical procedure compared to 21% of patients in the southern regions (*p* < 0.001) (Table 1).

There was a tendency for patients with glioblastoma in the southern regions to have a better WHO PS than those in the North, but there were no apparent differences in smoking status between the regions (Table 1).

For all groups of patients, there were no significant differences in the highest completed level of education or in cohabitation status between patients in the North and those in the southern regions (Table 1). The mean travel time to a regional hospital was longer for patients in the North for all groups, and low SES was associated with a longer travel time for all groups (Online Resource 2).

## Discussion

In this study, the multivariable survival analysis showed that patients with glioma WHO grade III-IV with the lowest SES (estimated through highest completed level of education) experienced the worst outcomes. However, no differences in survival according to travel time to a regional hospital, cohabitation status, or region of residence were found in the multivariable model for this population. For patients with lower-grade glioma (WHO grade I–II) and for patients with glioblastoma, neither SES, travel time to a regional hospital, cohabitation status, nor region were associated with survival.

The shorter survival time for patients with lower SES observed in this study is similar to the findings of Dalton et al., albeit those authors analyzed all types of CNS tumors together [5]. These differences in survival related to SES might be associated with other known disease-related prognostic factors, comorbidities, or access to health care.

A range of prognostic factors have been identified for patients with glioma WHO grade III and glioblastoma, including age, WHO PS, extent of surgery, postoperative treatment, O-6-methylguanine-DNA methyltransferase (MGMT) methylation status, and isocitrate dehydrogenase (IDH) mutation [26–34]. Dressler et al. studied sociodemographic factors and treatment decisions and found that old age, female sex, and low SES were associated with less access to full therapy [7], and in another study, longer waiting times for surgery were reported for glioma patients with lower SES [35]. In our multivariable model, SES was associated with survival despite adjusting for age and extent of surgery. Since data on WHO PS, postoperative treatment, MGMT methylation, and IDH mutation were missing in the study cohort, these variables were not included in the multivariable model.

In theory, access to care in Sweden´s publicly funded health care system should be equal to that of all citizens. However, actual access to care is affected not only by the availability of care but also by financial factors (affordability), health care-seeking behavior (acceptability), and geographic factors (accessibility) [36]. Health care-seeking behavior among patients with early cancer symptoms is associated with demographic factors, but a study by Svendsen et al. found no clear association with SES [37]. The present study did not include data on when or how glioma was diagnosed in patients. Consequently, differences in health care-seeking behavior might explain the disparity in survival according to SES in our study.

Longer travel times to the regional hospital were associated with older age and low SES in this study. In the multivariable analysis, no association between travel time and survival was found, which indicates that travel time to health care is not an independent prognostic factor for glioma. This study cannot conclude whether travel time impacts treatment decisions since treatment was not investigated in detail; however, no differences in survival related to travel time were observed in the multivariable analysis, which suggests that undertreatment was not related to travel time in this study cohort.

Cohabitation status was not associated with survival in our study, but earlier studies on cohabitation status and glioma survival have presented discordant findings. Dalton et al. found no difference in survival related to marriage for patients with CNS tumors, but Xie et al. found a better survival for glioblastoma patients who were married [5, 13]. Marriage might have a protective effect through improved mental, physical, and economic support and earlier diagnosis [13]. Cohabitant patients should benefit from similar social and physical support as married patients, and a favorable prognosis should appear stronger when studying cohabitation status instead of marriage. The lack of an association between cohabitation status and survival in our study might be explained by good access to support from health care for patients who are not cohabitants.

Finally, we found no difference in survival between the North and the southern regions despite several negative prognostic factors in the North, including higher age at diagnosis, a larger proportion of biopsies, and a tendency for worse WHO PS. We have no clear explanation for this finding. The quality registry data on age at diagnosis and surgery are most likely accurate. Reporting of WHO PS is more subjective, and it is possible that these data may not reflect a true difference between the regions. Other factors that could partly explain this finding include MGMT methylation, IDH mutation, postoperative treatment, and comorbidities.

### Strengths and limitations

This population-based study included all regions of Sweden with high coverage in the National Quality Registry for Brain Tumors. The access to health care and demographic registries in Sweden and the possibility of linking individual data from different sources using personal identity numbers represented another important strength of this study.

The primary limitation of this study was the lack of data in the main study cohort on comorbidities, lifestyle factors (smoking), prognostic factors (e.g., IDH mutation, 1p19q deletion, and MGMT methylation), and postoperative treatment. Molecular pathological analyses were not available, as the quality registry data were collected before the implementation of the WHO 2016 classification.

Estimation of SES through highest completed level of education might not always reflect true SES, and this was a potential limitation in this study.

## Conclusion

In this study of glioma prognosis, survival was significantly worse in patients with lower SES than in those with higher SES for WHO grade III-IV glioma. We found no significant differences in survival related to travel time to a regional hospital, cohabitation status, or region of residence (i.e., the North vs. the southern regions) in Sweden.

## Supplementary Information

Below is the link to the electronic supplementary material.

Supplementary file1 (DOCX 56 KB)

Supplementary file2 (DOCX 60 KB)

## Data Availability

The data analyzed in this study are available from the National Quality Registry for Brain Tumors, the LISA, the TPR, and the GD. Restrictions apply to the availability of these data, which were used under license for this study. Data are available from the authors upon reasonable request with the permission of the National Quality Registry for Brain Tumors, the LISA, the TPR, or the GD.

## Abbreviations

CNS: Central Nervous System
GD: Geography Database
ISCED: International Standard Classification of Education
LISA: Longitudinal Integration Database for Health Insurance and Labor Market Studies
PS: Performance Status
SES: Socioeconomic Status
TPR: Total Population Registry
WHO: World Health Organization
