# Supplementary material for: The impact of socioeconomic status on glioma survival: a retrospective analysis
Source: Cancer Causes Control. 2025 Jan 19;36(6):577–86. doi: 10.1007/s10552-025-01960-1 (PMC12098201; doi:10.1007/s10552-025-01960-1)
Supplement: Supplementary file 1 — Supplementary file1 (DOCX 56 KB) [file 10552_2025_1960_MOESM1_ESM.docx]

*The Impact of Socioeconomic Status on Glioma Survival: A Retrospective Analysis, Cancer Causes & Control. Maria Söderlund, Carl Almqvist, Olle Sjöström, Anna M Dahlin, Sara Sjöström, Barbro Numan Hellquist, Beatrice Melin, and Maria Sandström. Corresponding author: Maria Söderlund, Department of Diagnostics and Intervention, Oncology, Umeå University. SE-901 87 Umeå, Sweden, maria.el.eriksson@umu.se*

**Online Resource 1**

|  | **2009** | **2013** |
| --- | --- | --- |
| **Population^a^** |  |  |
| Total population in Sweden | 9,340,682 | 9,644,864 |
| Northern region | 877,308 | 878,706 |
| Three southern regions | 4,754,089 | 4,951,823 |

Population in Sweden categorized by health care region at the start and the end of the study period.

^a^ data from Statistics Sweden, <http://www.statistikdatabasen.scb.se/pxweb/en/ssd/>, accessed June 18, 2020
