# Supplementary material for: The impact of socioeconomic status on glioma survival: a retrospective analysis
Source: Cancer Causes Control. 2025 Jan 19;36(6):577–86. doi: 10.1007/s10552-025-01960-1 (PMC12098201; doi:10.1007/s10552-025-01960-1)
Supplement: Supplementary file 2 — Supplementary file2 (DOCX 60 KB) [file 10552_2025_1960_MOESM2_ESM.docx]

*The Impact of Socioeconomic Status on Glioma Survival: A Retrospective Analysis, Cancer Causes & Control. Maria Söderlund, Carl Almqvist, Olle Sjöström, Anna M Dahlin, Sara Sjöström, Barbro Numan Hellquist, Beatrice Melin, and Maria Sandström. Corresponding author: Maria Söderlund, Department of Diagnostics and Intervention, Oncology, Umeå University. SE-901 87 Umeå, Sweden, maria.el.eriksson@umu.se*

**Online Resource 2**

Mean travel time to a regional hospital in minutes.

|  |  | Glioma WHO grade I–II Mean travel time (n) | Glioma WHO grade III–IV  Mean travel time (n) | Glioblastoma (Glioma WHO grade IV)  Mean travel time (n) |
| --- | --- | --- | --- | --- |
|  |  |  |  |  |
| Region | Northern Sweden | 156.0 (50) | 169.5 (209) | 172.0 (176) |
|  | Southern regions | 52.2 (213) | 57.7 (804) | 56.6 (623) |
|  | *p-value ^a^* | *<0.001* | *<0.001* | *<0.001* |
|  |  |  |  |  |
| Sex | Men | 75.1 (134) | 80.6 (621) | 80.1 (490) |
|  | Women | 68.6 (129) | 81.1 (392) | 84.9 (309) |
|  | *p-value ^a^* | *0.50* | *0.92* | *0.45* |
|  |  |  |  |  |
| Age at diagnosis (years) | 0–39 | 64.9 (96) | 64.3 (58) | 70.8 (26) |
|  | 40–59 | 65.2 (102) | 74.4 (328) | 72.4 (251) |
|  | 60–69 | 97.7 (43) | 80.5 (341) | 81.3 (281) |
|  | ≥70 | 83.2 (22) | 91.7 (286) | 94.0 (241) |
|  | *p-value ^a^* | *0.080* | *0.042* | *0.046* |
|  |  |  |  |  |
| Education level | Middle school | 102.4 (45) | 94.0 (258) | 96.1 (211) |
|  | High school | 75.5 (106) | 85.7 (416) | 87.4 (319) |
|  | University/college | 55.3 (107) | 63.9 (332) | 64.2 (264) |
|  | *p-value ^a^* | *0.002* | *<0.001* | *<0.001* |

^a^ Differences between region and sex were assessed within each patient group by t test, and differences among age categories and education levels within each patient group were assessed by ANOVA.
